# Mechanism study on highly efficient polymer light-emitting diodes utilizing double-layered alkali halide electron injection layer

**DOI:** 10.1038/s41598-019-54729-3

**Published:** 2019-12-03

**Authors:** Qiaoli Niu, Jing Tong, Xiaomeng Duan, Haoran Zhang, Dexu Wang, Gang Hai, Hao Lv, Wenjin Zeng, Ruidong Xia, Yonggang Min

**Affiliations:** 1 Key Laboratory for Organic Electronics and Information Displays & Institute of Advanced Materials, Jiangsu National Synergetic Innovation Center for Advanced Materials (SICAM), Nanjing University of Posts and Telecommunications, 9 Wenyuan Road, Nanjing, 210023 P.R. China; 2 The School of Materials and Energy, Guangdong University of Technology, Panyu, Guangzhou, 510006 P.R. China; 3 New Energy Technology Engineering Laboratory of Jiangsu Province, Nanjing University of Posts and Telecommunications, Nanjing, 210023 Jiangsu China

**Keywords:** Conjugated polymers, Organic LEDs

## Abstract

Enhancing the injection of electron is an effective strategy to improve the performance of polymer light-emitting diodes (PLEDs). In this work, we reported a 286% improvement in current efficiency (CE) of PLEDs by using double-layered alkali halide electron injection layer (EIL) NaCl/LiF instead of LiF. A significant enhancement of electron injection was observed after inserting the NaCl layer. To understand the mechanism of such improvement, the devices with KBr/LiF and CsF/LiF as EILs were also investigated. Experimental results show that metal cation migrated under the effect of built-in electric field (V_bi_), which plays the main role on the improvement of electron injection in PLEDs.

## Introduction

Polymer light-emitting diodes (PLEDs) have absorbed tremendous attentions all over the world because of their attractive application potentials in flat panel display and solid-state lighting^1–3^. Although great progresses have been made on the performance of PLEDs since the first report of efficient PLEDs, there are still some issues have to be addressed^4–6^. One of the urgent issues is to realize efficient electron injection^7,8^ to achieve the balance of electron and hole current, which will lead to the enhancement of PLEDs performance^9^.

So far, many methods were selected to improve the electron injection of PLEDs. Low-work-function metals or alloys, such as Ba, Ca, Mg: Ag, Li: Al were used to lower the energy barrier of electron injection^4,10–14^. Interfacial dipole layer at the interface of emissive layer (EML) and cathode was used to elevate the vacuum energy level of cathode^15^, which can be formed by inserting interface layer such as ionic liquid^16^ and conjugated polyelectrolyte^17–20^, or by modifying the surface of EML with polar solvents such as DMF, methanol, ethanol, and 2,2,3,3,4,4,5,5-octafluoro-1-pentanol (F-alcohol)^21–23^. Alkali halide such as CsF and LiF is also commonly used electron injection materials (EIMs)^24,25^.

In addition of the methods mentioned above, the combination using of EIMs as EILs is also an efficient strategy, such as polyelectrolyte/Ba^26^, polar solvent/LiF^22^, LiF/Ca^27^, NaCl/Ca^28,29^, CsF/Ca^30^, and CsF/Yb^31^. Some possible mechanisms were raised to explain the improvement of electron injection in the case of the combination using of alkali halide and low-work-function metal. One was the combined effect of low-work-function metal such as Ba, Ca and alkali metal Cs or Li from evaporated CsF^30^ or LiF^32^. The second possible mechanism was the formation of interface dipole layer because of the molecular dipole properties of CsF or LiF^33^. The last possible mechanism was the diffusion of alkali halide^34^ or alkali atom^35,36^. However, in this work, we found an additional work mechanism of double-layered EIL based on alkali halide in PLEDs.

In this work, NaCl/LiF were used as the EIL in PLEDs, and a 286% improvement in current efficiency (CE) compared with the control device with LiF as EIL were achieved. The improvement of electron injections was responsible for the performance enhancement of PLEDs. To understand the working mechanism of NaCl/LiF, PLEDs based on KBr/LiF and CsF/LiF EILs were also studied. The mechanism investigation suggested that instead of the diffusion of alkali metal atoms reported previously, the drift of alkali metal ions under the effect of build-in electric field (V_bi_) of PLEDs was the main reason for the improvement of electron injection. Our study provides a simple approach to realize highly efficient electron injection and a basis for understanding the mechanism of multi-layered EILs in PLEDs.

## Results

PLEDs with device configurations of ITO/PEDOT:PSS (30 nm)/P-PPV(70 nm)/EIL/Al were fabricated by using NaCl/LiF, KBr/LiF and CsF/LiF as EIL, respectively. NaCl, KBr and CsF layers were prepared by spin-coating their solutions in methanol. The solution concentration was optimized according to the CE values of PLEDs, as shown in Fig. S1. In consideration of the effect of pure methanol on the electronic energy level structure at the interface of EML and cathode, device based on methanol treated P-PPV was also fabricated by spin-coating pure methanol on the surface of P-PPV. Details of device fabrication and performance measurements can be found in the experimental section.

**Figure 1:**
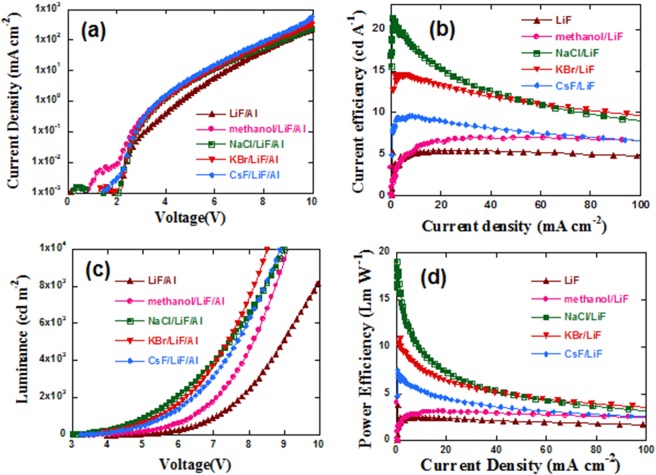
(**a**) Current density-voltage curves, (**b**) current efficiency-current density curves, (**c**) luminance-voltage curves, and (**d**) power efficiency-current density curves of PLEDs.

Figure 1 shows the current density-voltage-luminance (J-V), current efficiency-current density (CE-J) curves, luminance-voltage curves (L-J), and (d) power efficiency-current density curves (Lm/W) of the PLEDs with and without methanol, NaCl, KBr and CsF. The detailed performance parameters are summarized in Table 1. The control device with LiF as the EIL has a peak CE of 5.46 ± 0.08 cd/A and a peak external quantum efficiency (EQE) of 2.18 ± 0.03%. After inserting NaCl, KBr, and CsF layer, the CE values increased significantly to 21.05 ± 1.47, 14.49 ± 0.21 and 9.48 ± 0.76 cd/A, respectively, that is 286%, 165% and 74% increase compared with the control device. Meanwhile, the electroluminescence (EL) spectra (Fig. S2) of all the PLEDs mentioned above were the same, which demonstrated that the insertion of NaCl, KBr and CsF did not influence the energy state of the excited electron of P-PPV^21^. The increase of CE values is attributed to the improvement of EQE, which were 8.41 ± 0.66%, 5.81 ± 0.08% and 3.79 ± 0.31% for NaCl, KBr and CsF based PLEDs, respectively.

**Table 1:** The performance parameters of PLEDs.

EIL	V_th_^a^ (V)	Peak EQE^b^ (%)	Peak CE^c^ (cd A^−1^)	Peak Luminance (cd m^−2^)	Peak CE		
					@V (V)	@J (mA cm^−2^)	@L^d^ (cd m^−2^)
LiF	3.96 ± 0.16	2.18 ± 0.03	5.46 ± 0.08	19874	7.32	24.3	1328
methanol/LiF	3.72 ± 0.48	2.94 ± 0.22	7.34 ± 0.55	29439	7.44	35.06	2573
NaCl/LiF	2.64 ± 0.02	8.41 ± 0.66	21.05 ± 1.47	24453	4.2	2.11	444
KBr/LiF	2.88 ± 0.03	5.81 ± 0.08	14.49 ± 0.21	28078	5.4	6.62	959
CsF/LiF	2.88 ± 0.04	3.79 ± 0.31	9.48 ± 0.76	18968	5.16	6.66	631

^a^V_th_ is defined as the voltage at 1 cd m^−2^; ^b^EQE is refer to external quantum efficiency; ^c^CE is refer to the current efficiency; ^d^L is refer to luminance.

In addition, for the PLED based on methanol treated P-PPV, the CE and EQE values increased by 34% (7.34 ± 0.55 cd/A and 2.94 ± 0.22%) compared with the control device, as shown in Fig. 1 and Table 1, which are in good agreement with the previous report (37% increase)^22^. However, the magnitude of increase was much lower compared with PLEDs using NaCl/LiF, KBr/LiF or CsF/LiF as EILs. Thus, the enhancements of PLEDs performance by inserting NaCl, KBr and CsF were mainly caused by the alkali halides rather than the methanol solvent. PLEDs based on NaCl/LiF achieved the maximum CE and EQE values among the above alkali halides contained devices.

We noticed that the current densities in Fig. 1 increased after the insertion of NaCl, KBr and CsF. Meanwhile, the corresponding turn on voltage (V_th_), which is refer to the voltage at a luminance of 1 cd m^−2^, decreased from 3.96 ± 0.16 V of the control device to 2.64 ± 0.02 V, 2.88 ± 0.03 V, and 2.88 ± 0.04 V, respectively. Therefore, we speculated that the electron injection of PLEDs was enhanced.

**Figure 2:**
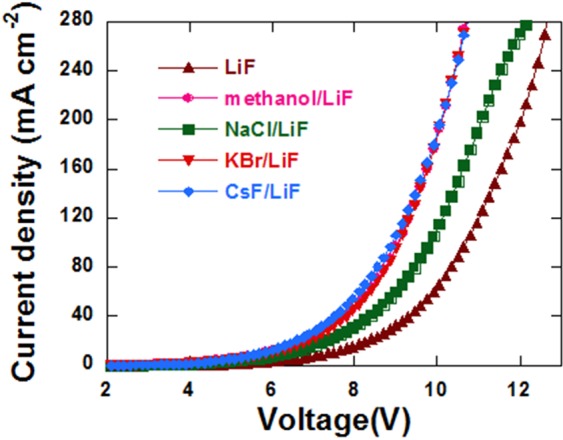
J-V curves of electron-only devices with device configurations of ITO/TiO_2_/P-PPV/EIL/Al.

In order to study the injection of electron, electron-only devices with configuration of ITO/TiO_2_/P-PPV/EIL/Al were fabricated. The J-V curves are shown in Fig. 2. Under a certain applied voltage, the control device had the smallest current density, which increased significantly after inserting NaCl, KBr and CsF. Although both the enhancement of electron injection and mobility can increase the electron current, the decrease of V_th_ indicates the improvement of electron injection.

**Figure 3:**
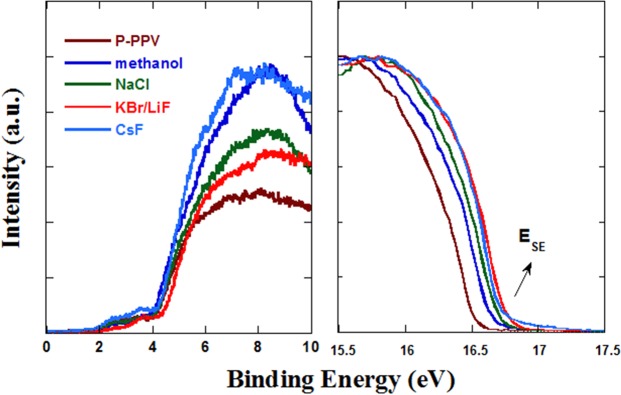
UPS spectra of P-PPV without and with treatment.

The electron injection of PLEDs are always influenced by the work function of the cathode^37^. Ultraviolet Photoelectron Spectroscopy (UPS) was commonly used to detect the surface electron energy level structure of material. Therefore, the UPS spectra on the surface of P-PPV with or without methanol, NaCl, KBr and CsF were collected, as shown in Fig. 3. The secondary electron cut-off (E_SE_) values were summarized in Table 2. After methanol treatment, the E_SE_ value increased from 16.51 to 16.64 eV, which is in accordance with the previous reports^21,22^. That caused by the formation of dipole layer between P-PPV and LiF because of the polarity of methanol^21,22^. The value increased further to 16.7, 16.75 and 16.72 eV with NaCl, KBr and CsF, respectively. It indicated that the vacuum energy level at the surface was lifted about 0.2 eV compared with the pristine P-PPV film (16.51 eV). Therefore, the electron injection barrier from LiF/Al to P-PPV reduced by 0.2 eV.

**Table 2:** The E_SE_ values of P-PPV films without and with treatment.

Film	P-PPV	P-PPV/methanol	P-PPV/NaCl	P-PPV/KBr	P-PPV/CsF
E_SE_ (eV)	16.51	16.64	16.7	16.75	16.72

**Figure 4:**
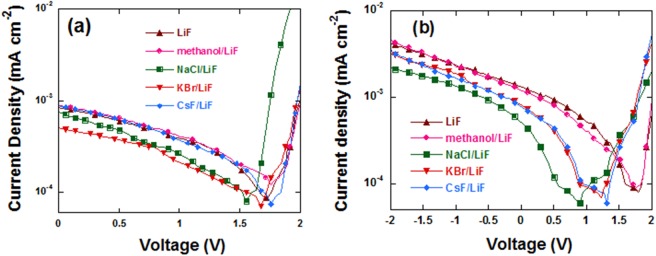
J-V curves of PLEDs under a simulated AM 1.5 illumination (100 mW cm^−2^), (**a**) under reverse scan from 2~0 V, and (**b**) under forward scan from −2~2 V.

In order to further study the reasons for the improvement of device performance by NaCl, KBr and CsF, the V_bi_ of PLEDs were measured. The J-V curves under an illumination of 1 sun (100 mW/cm^2^ AM 1.5 G) are shown in Fig. 4, and the detailed V_bi_ values are summarized in Table 3. V_bi_ in PLEDs can be used to estimate the work function difference between the anode and cathode. According to the results of UPS data, we expected an increase of V_bi_ values with the insertion of NaCl, KBr or CsF because of the elevation of vacuum energy level at the cathode. However, Fig. 4(a) and Table 3 show that the V_bi_ values decreased from 1.7 eV of the control device to 1.56 eV and 1.68 eV for the device with NaCl and KBr, respectively. In consideration of the vacuum energy level elevation at the surface of P-PPV, the decrease of V_bi_ values with NaCl or KBr could be caused by the work function change at the anode side due to the migration of Na, K and Cs from the cathode toward anode. V_bi_ in PLEDs were formed because of the work function difference between the anode and cathode, which goes from cathode to anode. Therefore, the positive ions at cathode will migrate along the direction of V_bi_ from cathode to anode. Thus, Na^+^, K^+^ and Cs^+^ migrated toward the anode of PLEDs under the effect of V_bi_. Na^+^, K^+^ and Cs^+^ are low-work function metal ions, whose migration toward the anode reduced the V_bi_ of PLEDs. That is to say, the work function of the anode may be lowered. It had been proven that the work function of the anode was lowered by treating the surface of PEDOT:PSS with polar solvent method or ethanol, leading to the decrease of hole current. We also observed the decrease of hole current after treating the surface of PEDOT:PSS with NaCl, KBr and CsF, respectively. The device configuration of the hole-only device is ITO/PEDOT:PSS/alkali metal halides/P-PPV/Al, whose J-V curves are shown in Fig. S3. It indicated that the work-function of the anode could be reduced because of the Na^+^, K^+^ and Cs^+^ cations at the anode, which can also be introduced through migration after treating the surface of P-PPV using NaCl, KBr and CsF. Because of the reduction of hole current, the balance of electron and hole current of PLEDs can be improved. Thus, the migration of Na^+^, K^+^ and Cs^+^ towards anode is benefit to the performance of PLEDs. Meanwhile, the doping of EML caused the little faster efficiency roll-off than that of the control device, which should be improved in the following work.

**Table 3:** Detailed V_bi_ values of PLEDs.

V_bi_	LiF	methanol/LiF	NaCl/LiF	KBr/LiF	CsF/LiF
reverse scan (2~0 V) (eV)	1.70	1.75	1.56	1.68	1.76
forward scan (−2~2 V) (eV)	1.72	1.78	0.91	1.23	1.31
series resistance (Rs) (Ω cm)	1.15 × 10^7^	1.89 × 10^7^	1.02 × 10^8^	7.63 × 10^7^	2.65 × 10^7^

To verify the migration of Na^+^, K^+^ and Cs^+^, forward scan from −2~2 V were applied on PLEDs when collecting the V_bi_ data. As shown in Fig. 4(b) and Table 3, the control device had the largest V_bi_ value, which decreased to 0.91, 1.23 and 1.31 eV after the insertion of NaCl, KBr and CsF, respectively. Different from the reverse scan (2-0 V), the voltage from −2~0 V was applied first in the case of the forward scan (from −2~2 V). The reverse bias was in the same direction with V_bi_, which was in favor of the migration of Na^+^, K^+^ and Cs^+^ ions. Therefore, V_bi_ values decreased greater than that in the reverse scan case, in which only positive voltage was applied. This verified our speculation about the irons migration under V_bi_ rather than the diffuse of their atom counterpart as reported previously^34–36,38^. In addition, among the double EILs devices, the NaCl based device shows the largest V_bi_ difference between the forward scan and reverse scan. This is due to the smallest atomic diameter of Na among Na, Br and Cs, which was in favor of the migration of ions. Though the atomic diameter of Li^+^ was smaller than Na^+^, the V_bi_ difference between the forward scan and reverse scan of the PLED based on LiF/Al is negligible. This may be because that Li reacted with Al during the evaporation process^39^. We can concluded that evaluation of vacuum energy level on the surface of P-PPV and the migration of low-work-function metal cations Na^+^, Br^+^ and Cs^+^ were both responsible for the enhancement of electron injection^34–36,38^.

In addition, during the V_bi_ test, the series resistance (R_s_) of the devices were also recorded as summarized in Table 3. We can see that the NaCl based device had the largest R_s_ value, which was almost one order of magnitude larger than that of the other devices. Thus the current density of NaCl based PLEDs was lower than the other cathode modified PLEDs, but still larger than that of the control device.

**Figure 5:**
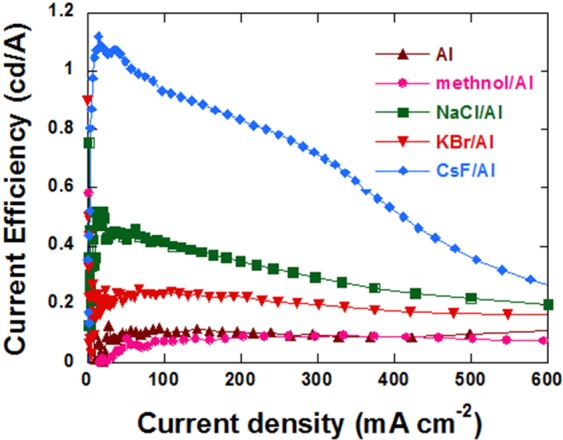
The CE-J curves of PLEDs with different EIL.

To further investigate the roles of LiF in the double-layered alkali metal halides EILPLEDs, Al cathode PLEDs with and without NaCl, KBr and CsF were fabricated. The CE-J curves were shown in Fig. 5 and the detailed performance parameters were summarized in Table 4. Figure 5 and Table 4 show that the peak CE values of PLEDs increased after the insertion of NaCl, KBr or CsF between P-PPV and Al cathode. However, CE values of all devices were much lower than that of the control device with LiF/Al cathode (5.46 ± 0.08 cd/A) as shown in Fig. 1 and Table 1. In addition, the V_th_ values of PLEDs with NaCl/Al or KBr/Al cathode were higher than that of the LiF/Al device, indicating the higher electron injection barrier. Though the deposition of NaCl or KBr on P-PPV lifted the vacuum energy level at the surface, the migration of Na^+^ and K^+^ toward the anode in PLEDs will reduce their effect on the surface, leading to the high electron injection barrier. The migration of Cs^+^ was difficult compared with Na^+^ and K^+^. Therefore, the V_th_ value of the CsF/Al device was lower than that of the NaCl/Al and KBr/Al devices. The lower efficiency of the CsF/Al device than that of the LiF/Al cathode could be caused by the different deposition method of LiF and CsF in this work. In the case of the PLEDs with double halide layers, the non-migrating Li^+^ at the cathode ensures the efficient electron injection. The incorporation of Na^+^ further reduced the electron injection barrier and improved the balance between electron and hole current. Therefore, NaCl/LiF EIL are more effective than LiF or NaCl in improving the performance of PLEDs.

**Table 4:** The detailed performance parameters of PLEDs with different EIL.

Cathode	V_th_ (V)	Peak EQE (%)	Peak CE (cd A^−1^)	Peak Luminance (cd m^−2^)	Peak CE		
					@V (V)	@J (mA cm^−2^)	@L (cd m^−2^)
Al	6.0	0.048	0.12	1112	8.76	94.2	112.2
methanol/Al	4.56	0.085	0.21	1720	7.44	93.6	192.8
NaCl/Al	4.2	0.207	0.52	1233	6.48	20.1	103.7
KBr/Al	4.32	0.107	0.25	1253	7.68	67.5	168.7
CsF/Al	2.76	0.448	1.12	2191	3.72	14.3	159.7

At last, the surface morphology of P-PPV with or without modification were also investigated by AFM. AFM height, phase and 3D images were shown in Fig. S3. We can see that there was no obvious change in considering of the morphology of P-PPV. The root mean square roughness (rms) values were 0.834 nm, 1.05 nm, 0.695 nm, 0.497 nm and 1.29 nm for the pristine film and methanol, NaCl, KBr and CsF treated films, respectively. After modification, the rms values had only slight difference, which had no obvious influence to the device performances.

## Discussion

We reported a 286% CE enhancements of PLEDs by using NaCl/LiF as the EIL instead of LiF. With NaCl on P-PPV, the vacuum energy level at the surface was lifted. The alkali metal ions migrated under the effect of build-in electric field from the cathode toward anode. Thus, electron injection was improved, leading to the enhancement of PLEDs performance. NaCl was more effect than KBr or CsF when used together with LiF as the EIL due to the maximum migration of Na^+^.

## Methods

### Materials and reagents

NaCl was purchased from Guoyao. KBr, and LiF were purchased from Macklin. CsF and methanol were both purchased from Sigma-Aldrich. Poly(2-(4-(3′,7′-dimethyloctyloxyphenyl)- 1,4-phenylene vinylene) (P-PPV) was purchased from Canton OLEDKING Optoelectric Materials Co. Ltd. Poly(3,4-ethylenedioxythiophene)-poly(styrenesulfonate) (PEDOT: PSS) was purchased from Xi’an Polymer Light Technology Corp. All the above materials were used as received.

### Polymer light-emitting diodes Fabrication

Control PLEDs with device structure of ITO/PEDOT:PSS (30 nm)/P-PPV (70 nm)/LiF(1 nm)/Al (150 nm). The detailed fabrication process can be found in our previous report^22^. For PLEDs based on NaCl, KBr or CsF, 1 mg/mL NaCl, KBr or CsF solutions in methanol or pure methanol solvent was spin-coated on P-PPV at a speed of 2000 rpm before the evaporation of LiF.

The device characterizations details can also be found in our previous report^22^.

## Supplementary information

 Supplementary Figures
