# Supplementary material for: Mechanism study on highly efficient polymer light-emitting diodes utilizing double-layered alkali halide electron injection layer
Source: Sci Rep. 2019 Dec 3;9:18232. doi: 10.1038/s41598-019-54729-3 (PMC6890694; doi:10.1038/s41598-019-54729-3)
Supplement: Supplementary file 1 — Supplementary Figures [file 41598_2019_54729_MOESM1_ESM.docx]

**Mechanism study on highly efficient polymer light-emitting diodes utilizing double-layered alkali halide electron injection layer**

Qiaoli Niu^1,3^, Jing Tong^1^, Xiaomeng Duan^1^, Haoran Zhang^1^, Dexu Wang^1^, Gang Hai^1^, Hao Lv^1^, Wenjin Zeng^1,3^, Ruidong Xia^1^* and Yonggang Min^2^*

^1^ Key Laboratory for Organic Electronics and Information Displays & Institute of Advanced Materials, Jiangsu National Synergetic Innovation Center for Advanced Materials (SICAM), Nanjing University of Posts and Telecommunications, 9 Wenyuan Road, Nanjing 210023, P.R. China.

^2^The School of Materials and Energy, Guangdong University of Technology, Panyu, Guangzhou 510006, P. R. China.

^3^New Energy Technology Engineering Laboratory of Jiangsu Province，Nanjing University of Posts and Telecommunications, Nanjing, 210023, Jiangsu, China.


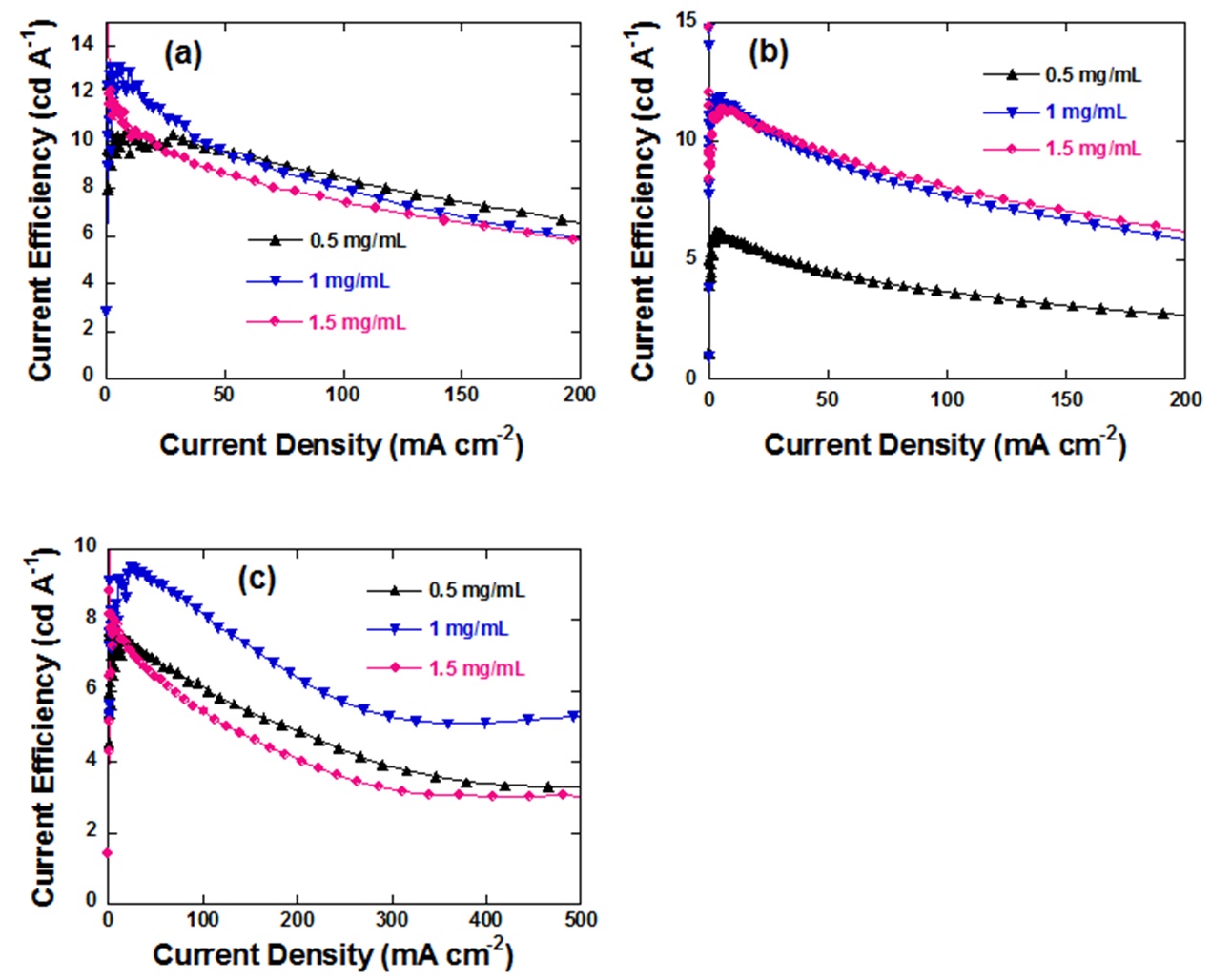


Fig. S1. The CE-J curves of PLEDs based on NaCl (a), KBr (b), and CsF (c), respectively, spin-coated from different concentrations in methanol solution.

Fig. S2. EL spectra of PLEDs with different EILs .

Fig.S3. J-V curves of hole-only devices with device structure of ITO/PEDOT:PSS/alkali metal halides/P-PPV/Al.


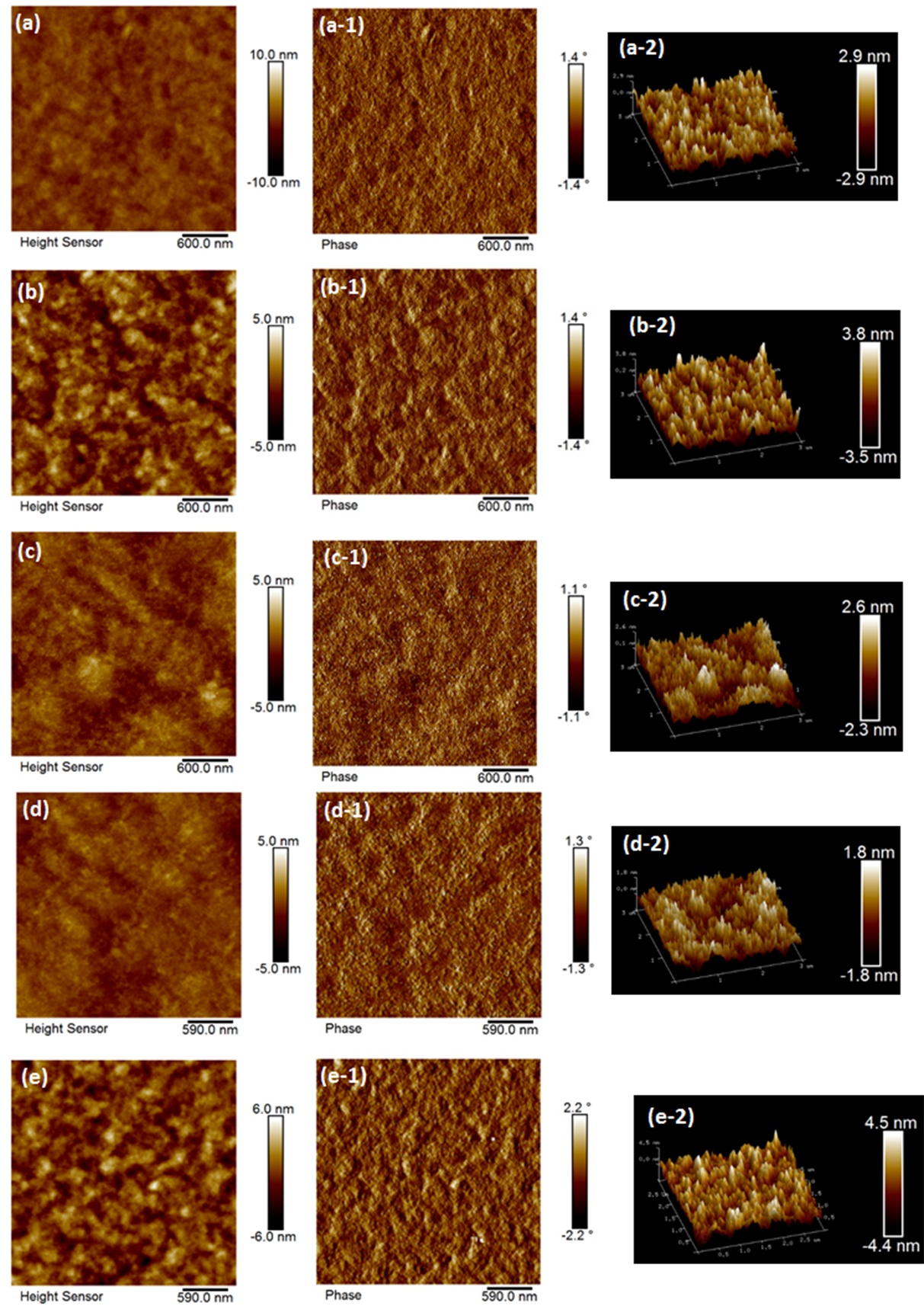


Fig. S4. AFM height, phase and 3D images of pristine P-PPV (a, a-1, a-2), methanol treated P-PPV (b, b-1, b-2), P-PPV/NaCl (c, c-1, c-2), P-PPV/KBr (d, d-1, d-2) and P-PPV/CsF (e, e-1, e-2).
